# Health-related quality of life in outpatients with substance use disorder: evolution over time and associated factors

**DOI:** 10.1186/s12955-022-01935-9

**Published:** 2022-02-16

**Authors:** Melexima Simirea, Cédric Baumann, Michael Bisch, Hélène Rousseau, Paolo Di Patrizio, Sarah Viennet, Stéphanie Bourion-Bédès

**Affiliations:** 1 Centre Psychothérapique de Nancy, CSAPA (Health Care Centre of Accompaniment and Prevention in Addictology), 54 520, Laxou, France; 2 UMDS (Unit of Methodology, Data Management and Statistics), University Hospital of Nancy, 54500 Vandoeuvre-lès-Nancy, France; 3 EA4360 APEMAC (Health Adjustment, Measurement and Assessment, Interdisciplinary Approaches) MICS Team, University of Lorraine, 54500 Vandoeuvre-lès-Nancy, France; 4 Centre Hospitalier de Versailles, Service Universitaire de Psychiatrie de l’Enfant et de l’Adolescent, 78150 Versailles, France

**Keywords:** Health-related quality of life, Outpatient care, Substance dependence, Follow-up

## Abstract

**Background:**

Health-related quality of life (HRQoL) is an important element of patient care and clinical research. The aim of this study was to describe HRQoL changes and identify associated factors during a 6-month follow-up of outpatients starting care for alcohol or opioid dependence.

**Methods:**

HRQoL was measured at baseline and 3 and 6 months later using the SF-12. Data on the patients’ sociodemographics, clinical characteristics and levels of anxiety and depression were collected using the Hospital Anxiety and Depression Scale (HADS). Repeated-measures analyses were performed to assess factors associated with global HRQoL differences and the evolution of HRQoL indicated by both physical and mental scores (PCS and MCS, respectively).

**Results:**

The mean PCS and MCS scores were initially low at 45.4 (SD = 8.6) and 36.0 (SD = 10.9), respectively. The improvement in HRQoL was rapid in the first 3-month period and then slowed and remained stable over the subsequent 3-month period. Being employed (*p* = 0.012), having no comorbidities (*p* = 0.014) and having no depression (*p* = 0.004) were associated with significant differences in the average PCS scores at the 3 time points. Patients who had lower overall HRQoL MCS scores on average were those for whom a medication was initiated (*p* = 0.009), as was the case for patients with anxiety (*p* < 0.001) and depression (*p* < 0.001). Patients with depression at baseline were also those for whom a significantly greater increase in MCS score during the 6 months of follow-up was observed.

**Conclusion:**

Our findings highlight the importance of screening early psychological distress and considering other factors associated with HRQoL changes in outpatients after the first 3-month period of treatment for substance use disorder.

## Introduction

Substance use disorder (SUD) produces unstable life patterns, as it impacts many areas of an individual’s global functioning across a broad range of life domains [1, 2]. SUDs adversely affect the quality of life of patients, including their working life, interpersonal relationships, social activities, and physical and mental states [3, 4]. Based on the Global Burden of Disease (GBD) study, substance abuse was responsible for 20 million disability-adjusted life years at the global level in 2010 [5]. Over the last two decades, self-report measures describing patients’ perspectives and experiences of living have been developed and included in studies examining new interventions or models of care in study participants [6]. There has been an increased interest in quality of life as an outcome measure in the field of substance abuse care [7–9], and patient-perceived health-related quality of life (HRQoL) has become an important and acknowledged indicator of treatment effectiveness, patient care/management and recovery in patients with SUD [10–12]. It has been shown that HRQoL is consistently low among individuals with SUD who actively seek treatment compared with individuals without SUD or those with chronic psychiatric conditions [13]. Longitudinal studies help to examine HRQoL as well as positive and significant changes during care in both men and women [14–16].

Follow-up assessments from previous studies showed that the improvement in HRQoL among outpatients with substance use disorders receiving care was rapid in the first 3 months [4, 16–18] and slower over the later period of 3–8 months when HRQoL assessments were repeated [18, 19]. A previous work even reported that the best HRQoL was achieved at 6 months of care during a 1-year observation period [20]. The results from longitudinal studies using the SF-36 or the SF-12 with substance use clinical populations reported more improvement over time, especially in patients’ mental states [17, 21]. Divergent findings were reported in patients’ physical states; some studies showed small but significant improvements in physical component summary scores [4, 22], whereas others did not [21].

Although the literature has shown that specialized SUD treatment enhances HRQoL for dependent patients [11], findings on the factors associated with changes in HRQoL are lacking [23]. However, identifying the predictors of HRQoL changes using longitudinal studies would inform clinicians on how to improve HRQoL by offering more improvement strategies as appropriate interventions during the process of care and thus enabling health planners, administrators and policy-makers to plan and deliver more effective and efficient care [24, 25]. Some studies revealed that QoL improved with substance abstinence [22], whereas others showed that there was no correlation between a reduction in substance use and HRQoL [26]. In addition, a published study found that sociodemographic and clinical factors, such as marital status, income and somatic comorbidities, explained differences in HRQoL changes [27]. Conflicting findings have been reported regarding the relationship between HRQoL and the presence of other mental disorders [28]. Some previous works have shown that comorbidities impact HRQoL [7, 29], while other authors did not find relationships between HRQoL and comorbidities and associated impaired HRQoL with variables related to patterns of drug use [21, 30].

Although there are several studies that assess HRQoL in the field of addiction research, there are few evaluating its evolution over time using a longitudinal design in outpatient settings. Indeed, in addition to inpatient settings, ambulatory care has increasingly become a focus among researchers aiming to improve addiction care for patients with substance use disorders [3, 31]. Therefore, this follow-up study aimed to assess dependent outpatients’ HRQoL changes at 3 and 6 months after initiating outpatient care and to explore the factors associated with HRQoL evolution during this 6-month follow-up period.

## Methods

### Study population

Data for this study were derived from the SUBstance Users Satisfaction and Quality Of Life (SUBUSQOL) study, a prospective multicentric cohort study on HRQoL and satisfaction with care in substance dependence (ClinicalTrials.gov ID: NCT02894476). Inclusion criteria were being aged over 18; having alcohol dependence or opioid dependence according to the Diagnostic and Statistical Manual of Mental Disorders, fourth edition (DSM-IV) [32]; and beginning care in one of four French specialized addiction treatment investigation centers. The participants were recruited by clinicians who were certified in treating addiction pathologies and were familiar with the DSM-IV. Treatment included individual motivation enhancement, supportive therapy, pharmacotherapy and assessments of somatic and mental health performed by multidisciplinary staff, including psychiatrists, psychologists, social workers and specialized nurses.

### Data collection

**Fig. 1:**
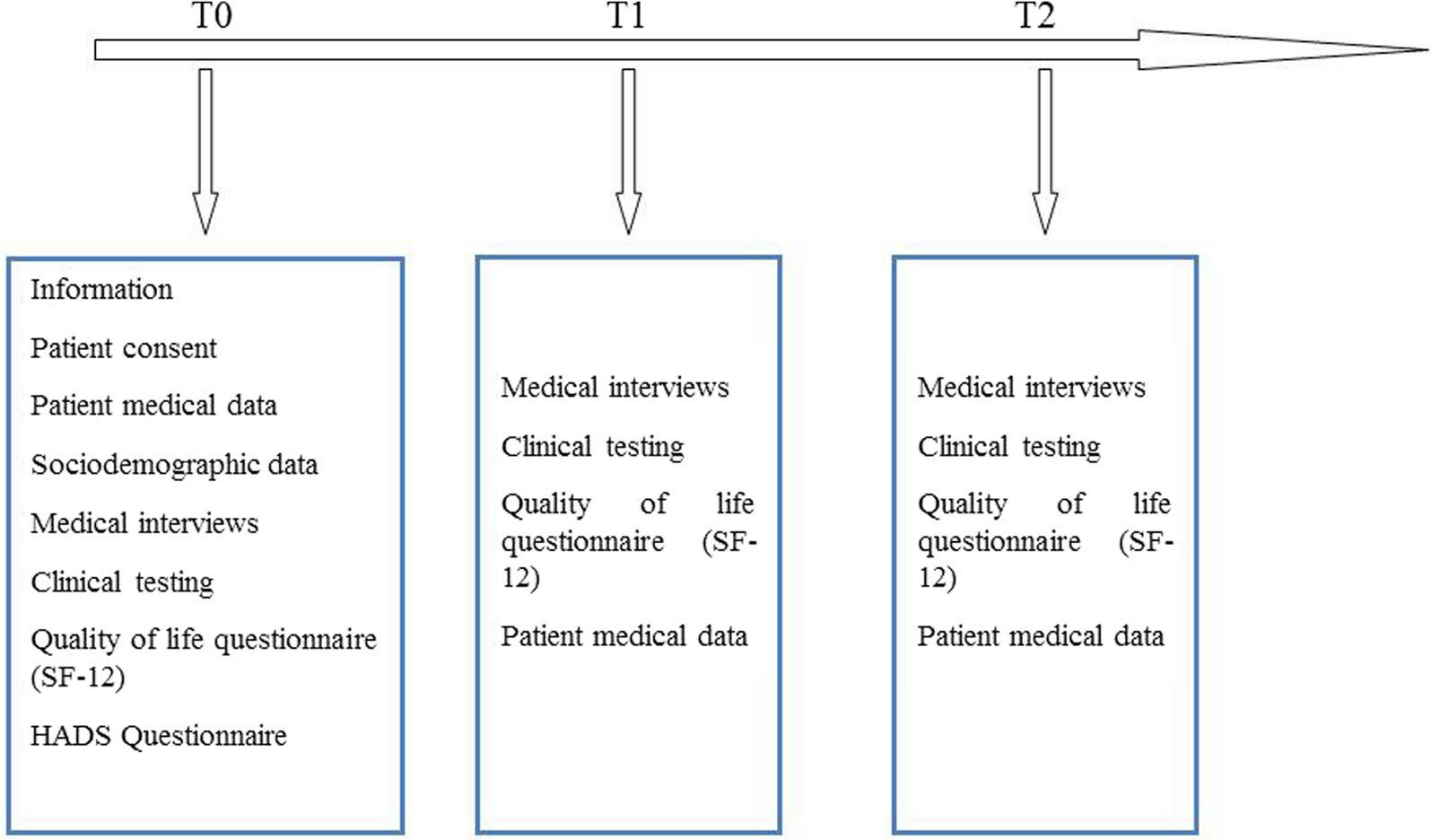
Time points of collection of SUBUSQOL data

Sociodemographic data were collected at study inclusion (T0), and clinical data were collected at inclusion (T0), 3 months (T1) and 6 months (T2) via medical interviews and clinical testing. Self-report questionnaires were used to assess anxiety and depression levels at T0 and self-reported quality of life at T0, T1 and T2 (Fig. 1).

#### Sociodemographic and medical data

Patients’ sociodemographic and medical characteristics were collected, such as sex, age, marital status, educational level, occupational status, type of substance dependence, duration of illness, presence of psychiatric and/or somatic comorbidities, medications introduced and origin of the care request. Data related to the physician, including sex, academic qualifications and years of clinical practice, were also reported.

#### Health-related quality of life

Health-related quality of life was assessed by the Medical Outcomes Study Short-Form 12-item Health Survey (SF-12) [33]. The questionnaire was completed as part of routine care at study inclusion and 3 and 6 months later. The SF-12 includes a subset of 12 items from the earlier SF-36 that covers eight domains: physical functioning, role-physical (that is, role limitations due to physical problems), bodily pain, general health, vitality, social functioning, role-emotional (that is, role limitations due to emotional problems) and mental health [34]. The French version yields valid and reliable clinical assessments of self-reported quality of life among substance users [35]. A physical health component summary (PCS) and a mental health component summary (MCS) were calculated from all 12 items. The properties of the SF-12 were evaluated in the same population in a previous study [4]. All scores are transformed into standardized 0–100 scores. Higher scores indicated better self-reported health status.

#### Anxiety and depression

The French version of the Hospital Anxiety and Depression Scale (HADS) was used to assess symptoms of anxiety and depression [36]. The HADS consists of 14 items, with 7 items assessing the level of anxiety and 7 items assessing the level of depression [37]. Each item is rated on a 4-point Likert scale, and the total scores for both subscales range from 0 to 21. For each subscale, the score is obtained by summing the respective 7 items (the subscale scores range from 0 to 21). Each subscale has three ranges using cutoff scores indicating the severity of distress levels: 0–7 (noncases), 8–10 (mild severity), and 11–21 (moderate or severe severity) [38]. The HADS properties were evaluated in the same population in a previous study [4].

### Statistical analyses

#### Sample description analyses

Continuous variables were described by the means and standard deviations. Medians were used to dichotomize variables where applicable. Categorical variables were described by percentages.

#### Evolution of SF-12 scores over time

ANOVAs with two factors (patient and time) were used to compare HRQoL scores at 0, 3 and 6 months, and a Bonferroni correction was applied for 2 by 2 comparisons.

#### Factors associated with SF-12 scores over time

To analyze the associations between the sociodemographic and clinical data and HRQoL evolution after 6 months of care, bivariable and multivariable repeated-measures ANOVAs were used (within and between effects). Variables with a *p* value < 0.1 in bivariable analysis were candidates for the multivariable model. Beforehand, the lack of collinearity was verified. No selection procedure was applied in the multivariable model. Finally, Bonferroni tests were used to compare HRQoL scores 2 by 2. Eta squared (E^2^) was used to estimate the effect size of each parameter in the multivariable model according to the following formula: η^2^var1 = SSvar1/(SSsubject + SSError + SSvar1 + SSvar2 + …) with SS = sum of squares. η^2^ = 0.01 indicated a small effect; η^2^ = 0.06 indicated a medium effect; and η2 = 0.14 indicated a large effect [39].

Analyses were performed using SAS 9.4 (SAS Inst., Cary, NC, USA).

### Ethics approval and consent to participate

Ethics approval was granted by the Institutional Review Board (Comité National Informatique et Liberté DR-2013–156), and the confidentiality of the collected data was ensured. Potential participants were informed of the study’s purpose and aims and received guidance about how they could withdraw consent at any point. Consent forms were signed in the presence of a researcher and were kept in a file at the main study site.

## Results

### Patient characteristics

The patients’ sociodemographic and clinical characteristics are presented in Table 1. Overall, 126 patients were included in the study; 80.2% were men, and 19.8% were women. The mean age was 39.6 years (SD = 10.1). In total, 46.3% of the patients were single, 32.5% were married, and 21.2% were separated, divorced or widowed. Slightly less than one-third of the sample (32%) reported full-time employment, and 20% had a high school level of education. Of the sample, 81 (64.3%) patients suffered from opioid dependence, and 45 (35.7%) patients exhibited alcohol dependence according to the DSM-IV criteria. The mean duration of the disorder was 15.7 years (SD = 11.0), and 36.5% of the patients (n = 46) presented somatic and/or psychiatric comorbidities. The baseline mean HADS score for the depression subscale was 8.3 (SD = 4.3), and the baseline mean HADS score for the anxiety subscale was 10.5 (SD = 4.7). In total, 67.5% of the patients had moderate or severe anxiety, and 58.2% of the patients had moderate or severe depression.

**Table 1:** Participant's sociodemographic and clinical characteristics at baseline

Characteristics	Full sample N = 126	
	N	Mean (SD) or %
Age (years)		38.5 (10.2)
Gender		
Male	101	80.2
Female	25	19.8
Marital status (missing = 3)		
Never married	57	46.3
Married/live with a partner	40	32.5
Separated/divorced/widowed	26	21.2
Educational level (missing = 1)		
Primary school	12	9.6
Secondary school	88	70.4
High school/university	25	20.0
Living arrangements (missing = 2)		
Alone	43	34.7
Alone with children	10	8.1
With partner	16	12.9
With partner and children	24	19.4
With family or friends	28	22.6
Homeless	3	2.4
Occupational status (missing = 1)		
Unemployed/student	72	57.6
Full-time work	40	32.0
Part-time work	8	6.4
Retired	5	4.0
Type of dependence		
Alcohol dependence	45	35.7
Opioid dependence	81	64.3
Duration of addiction (years)	124	5.3 (5.5)
Comorbid axis I diagnosis (yes)	46	36.5
Origin of the care request		
Patient	96	76.2
Justice	14	11.1
Heath practioner	16	12.7
HADS Anxiety (missing = 6)	120	10.5 (4.7)
< 8	39	32.5
≥ 8	81	67.5
HADS Depression (missing = 4)	122	8.3 (4.3)
< 8	51	41.8
≥ 8	71	58.2
Patient-physician gender match (yes)	51	40.5
Medication initiated during 6-month follow-up (yes)	73	58.4
Change in substance use behavior at 6 months (yes)	103	82.4
Number of medical sessions during 6-month follow-up	105	13.9 (5.4)

*SD* standard deviation, *HADS* Hospital Anxiety and Depression Scale

Fourteen patients who received care were undergoing legally mandated addiction treatment. All of the physicians were currently working with patients with substance dependence, and 83 outpatients were screened by a junior physician (65.9%). In 40.5% of all cases, the patient and physician were of the same gender. After 6 months, the average number of medical sessions completed was 13.9 (SD = 5.4), and positive changes in substance use behavior were observed for 103 (82.4%) outpatients.

### Evolution of HRQoL over time

Scores on the SF-12 over time are shown in Table 2. At T0, the mean SF-12 scores were 45.4 (SD = 8.6) and 36.0 (SD = 10.9) for the PCS and MCS domains, respectively. The HRQoL scores improved significantly from T0 to T2 (< 0.001 for PCS and MCS after Bonferroni correction), and different patterns of changes emerged according to the HRQoL domain. The PCS scores improved between T0 and T1 and then reached a plateau by T2, while the MCS scores continued to increase from T0 to T2.

**Table 2:** Self-reported Health status scores over time

Self-reported Health status	Intake^0^	3 months^3^	6 months^6^	*p* value*
	Mean (SD) or %	Mean (SD) or %	Mean (SD) or %	
SF-12				
PCS score	45.4 (8.6)	48.4 (7.9)	48.4 (8.4)	< 0.0001^03/06^
MCS score	36.0 (10.9)	41.6 (11.3)	44.1 (11.3)	< 0.0001^03/06^

*SD* standard deviation; *SF-12*, short-form 12; *PCS*, physical component summary; *MCS*, Mental health Component Summary; N=126

*ANOVA at two factors : id and time

^XY^statistically significant 2 to 2 differences after Bonferroni correction

### Factors associated with average HRQoL during the 6-month follow-up

The associations between the sociodemographic and clinical data and the global average HRQoL during the 6-month period of care are presented in Tables 3 and 4. Table 3 shows significant associations between PCS scores and 3 variables: occupational status (E2 = 0.022), comorbidity axis I diagnosis (E2 = 0.021) and HADS depression score (E2 = 0.029). Patients who were employed (*p* = 0.012), those with no health problems other than their addictive disorder (*p* = 0.014) and those with no depression on the HADS questionnaire (HADS depression < 8) (*p* = 0.004) had an average higher overall HRQoL during 6 months of follow-up than the others. No factor interacted with time.

**Table 3:** Factors associated with HRQoL changes on the physical domain (SF 12-PCS) N = 126

Characteristics	Mean ± SD			Bivariable analysis	Multivariable analysis (R2 = 0.2)	
	T0	T1	T2	*p* value	*p* value	Effect size η^2^
Age				0.002	0.070	0.011
≤ 38.5	48.27 ± 6.91	50.11 ± 6.53	49.73 ± 6.85			
> 38.5	43.03 ± 9.27	46.68 ± 8.74	46.98 ± 9.60			
Gender				0.18		
Male	45.92 ± 8.33	48.76 ± 7.44	48.71 ± 8.30			
Female	43.45 ± 9.35	46.75 ± 9.41	47.03 ± 8.87			
Marital status				0.43		
Never married	45.27 ± 7.20	48.84 ± 7.78	47.84 ± 8.06			
Married/live with a partner	46.38 ± 8.58	49.52 ± 6.72	48.97 ± 8.00			
Separated/divorced/widowed	44.31 ± 11.10	45.53 ± 9.17	48.32 ± 9.78			
Educational level				0.77		
Primary school	42.75 ± 6.53	51.34 ± 6.35	48.65 ± 11.44			
Secondary school	46.28 ± 8.48	48.16 ± 7.96	48.51 ± 8.08			
High school/university	43.71 ± 9.57	47.88 ± 8.27	47.93 ± 8.40			
Occupational status				0.008	0.012	0.022
Unemployed	44.65 ± 8.85	46.76 ± 8.31	46.72 ± 8.74			
Employed	46.66 ± 8.12	50.74 ± 6.58	50.71 ± 7.21			
Type of dependence						
Alcohol dependence	44.80 ± 10.29	47.75 ± 8.48	47.78 ± 9.26	0.46		
Opioid dependence	45.78 ± 7.47	48.70 ± 7.55	48.71 ± 7.93			
Duration of addiction (years)				0.07	1.00	0.00
≤ 13	46.78 ± 8.10	48.98 ± 7.19	49.48 ± 7.36			
> 13	43.96 ± 8.95	47.63 ± 8.62	46.95 ± 9.23			
Comorbid axis I diagnosis				0.001	0.014	0.021
Yes	42.67 ± 9.18	45.38 ± 9.28	46.40 ± 9.16			
No	47.02 ± 7.80	50.08 ± 6.40	49.52 ± 7.77			
Origin of the care request				0.15		
Patient	44.16 ± 8.39	48.04 ± 8.22	48.07 ± 8.66			
Justice	51.83 ± 7.06	48.78 ± 5.80	49.90 ± 6.73			
Health practitioner	47.47 ± 8.23	49.95 ± 7.51	48.88 ± 8.46			
Medication initiated during 6-month follow-up				0.43		
Yes	45.27 ± 8.44	47.82 ± 7.47	47.98 ± 8.30			
No	45.68 ± 8.88	49.33 ± 8.33	49.06 ± 8.63			
Change in substance use behavior at 6 months				0.30		
Yes	45.33 ± 8.79	48.00 ± 7.99	48.10 ± 8.50			
No	45.95 ± 7.79	50.57 ± 6.86	49.94 ± 8.07			
HADS anxiety				0.01	0.34	0.003
< 8	48.85 ± 8.43	50.34 ± 6.77	50.53 ± 7.00			
≥ 8	44.20 ± 8.20	47.59 ± 8.30	47.78 ± 8.69			
HADS depression				0.001	0.004	0.029
< 8	48.21 ± 8.26	50.85 ± 6.15	50.25 ± 8.22			
≥ 8	43.38 ± 8.17	46.62 ± 8.53	47.23 ± 8.19			

*SD* standard deviation, *HADS* Hospital Anxiety and Depression Scale

*Significant interaction with time for HADS depression (*p* < 0.05)

**Table 4:** Factors associated with HRQoL changes on the mental domain (SF-12 MCS) N = 126

Characteristics	Mean ± SD			Bivariable analysis	Multivariate analysis (R^2^ = 0.08)	
	T0	T1	T2	*p* value	*p* value	Effect size η^2^
Age				0.50		
≤ 38.5	36.97 ± 10.59	42.38 ± 11.23	44.49 ± 11.44			
> 38.5	35.55 ± 11.77	41.19 ± 11.51	43.92 ± 11.23			
Gender				0.44		
Male	36.46 ± 11.48	42.20 ± 11.55	43.93 ± 11.52			
Female	33.94 ± 8.07	39.17 ± 10.03	44.945 ± 10.36			
Marital status				0.96		
Never married	36.13 ± 11.04	41.22 ± 11.02	44.09 ± 10.94			
Married/live with a partner	36.68 ± 10.12	42.48 ± 10.70	43.84 ± 11.37			
Separated/divorced/widowed	34.81 ± 12.15	42.08 ± 12.60	44.90 ± 12.16			
Educational level				0.24		
Primary school	41.76 ± 12.68	43.62 ± 12.43	46.52 ± 13.90			
Secondary school	35.74 ± 10.52	41.67 ± 10.91	44.50 ± 10.89			
High school/university	34.08 ± 11.13	40.76 ± 12.51	41.51 ± 11.44			
Occupational status				0.09	0.08	0.009
Unemployed	37.06 ± 11.39	42.32 ± 11.37	45.27 ± 10.86			
Employed	34.04 ± 9.96	40.32 ± 11.23	42.23 ± 11.81			
Type of dependence				0.88		
Alcohol dependence	35.66 ± 11.67	41.16 ± 11.96	44.39 ± 12.14			
Opioid dependence	36.13 ± 10.53	41.85 ± 10.98	43.99 ± 10.83			
Duration of addiction (years)				0.70		
≤ 13	36.08 ± 10.52	41.67 ± 10.88	45.06 ± 10.46			
> 13	36.08 ± 11.47	41.56 ± 11.70	43.35 ± 11.85			
Comorbid axis I diagnosis				0.66		
Yes	37.02 ± 11.31	40.20 ± 11.35	45.85 ± 10.90			
No	35.35 ± 10.69	42.40 ± 11.26	43.15 ± 11.42			
Origin of the care request				0.18		
Patient	36.64 ± 10.73	40.61 ± 11.58	43.29 ± 11.34			
Justice	38.25 ± 11.35	47.32 ± 10.57	47.23 ± 10.31			
Health practitioner	35.88 ± 12.04	42.53 ± 8.93	46.51 ± 11.48			
Medication initiated during 6-month follow-up				0.004	0.009	0.021
Yes	33.37 ± 10.02	39.67 ± 10.87	43.65 ± 11.57			
No	39.63 ± 11.25	44.77 ± 10.90	45.31 ± 10.36			
Change in substance use behavior at 6 months				0.13		
Yes	34.69 ± 10.39	41.27 ± 10.82	44.53 ± 11.06			
No	41.97 ± 11.76	44.23 ± 12.43	43.46 ± 11.33			
HADS anxiety				< 0.001	< 0.001	0.048
< 8	43.65 ± 10.19	48.40 ± 10.42	47.22 ± 10.74			
≥ 8	32.00 ± 9.08	38.66 ± 10.51	42.27 ± 11.40			
HADS depression				< 0.001	< 0.001	0.034
< 8	42.27 ± 10.85	45.17 ± 11.09	46.86 ± 10.32			
≥ 8	30.93 ± 8.04	39.12 ± 10.99	41.90 ± 11.66			

*SD* standard deviation, *HADS* Hospital Anxiety and Depression Scale

*Significant interaction with time for HADS depression (*p* < 0.05)

The results for MCS scores are presented in Table 4. Patients who had an average lower overall HRQoL score were those for whom medication was initiated during the 6-month follow-up (*p* = 0.009; E2 = 0.021), as was the case for patients with anxiety (*p* < 0.001; E2 = 0.048) and depression (*p* < 0.001; E2 = 0.034) at baseline according to the HADS.

### Factors associated with HRQoL changes over time at the 6-month follow-up

A single significant interaction with time was observed with the HADS depression score, indicating that a difference was found in the magnitude of change in the MCS score regardless of the HADS depression score at baseline. Patients with depression at inclusion were those for whom a significantly greater increase in MCS score during the 6 months of follow-up was observed.

## Discussion

In this 6-month follow-up study, we investigated longitudinal associations between patients’ sociodemographic, clinical and health-related status at baseline, and we examined HRQoL changes over time. First, the mean physical and mental domain scores for our study participants were lower than the PCS and MCS scores of the French general population, which have been reported to be 48.4 (SD = 9.4) and 51.2 (SD = 7.4), respectively [34]. This finding corroborates previous works, which showed that the HRQoL was consistently low among patients with SUD compared with the general population or individuals with other chronic health conditions [11, 13, 40]. According to previous findings suggesting that an MCS score < 42.0 indicates significant impairment [33, 41], the mental domain was particularly impaired in our study population. Second, consistent with other studies [17, 42] and given repeated assessments of generic HRQoL instruments, this study also found that improvements in HRQoL occurred rapidly in the first 3-month period and then slowed and became stable over the subsequent 3-month period [41, 43, 44]. In contrast to the findings about the relationship between treatment for SUD and changes in physical HRQoL [42], our study found significant improvement between the mean SF-12 physical component summary scores during care, particularly during the first 3 months. Significant improvement in the SF-12 physical component summary scores was observed during care, particularly during the first 3 months. Even if significant improvements in SF-12 scores were seen during care, both physical and mental scores at the 6-month follow-up were still below the French general norms [34].

Among the baseline sociodemographic characteristics, occupational status was the only parameter associated with an average higher overall HRQoL in the physical domain during the 6-month follow-up period. Regarding occupational status, it is well known that being employed is consistently associated with better HRQoL scores [45, 46]. In our study, neither length of substance dependence nor changes in alcohol and/or opioid consumption were significantly associated with quality of life functioning during the 6-month follow-up, in line with previous research [7, 47]. This result highlights that other factors may play an important role in the HRQoL for individuals with substance use disorders beyond just abstinence or a reduction in substance use and that a restricted focus on drug-related issues will have only a limited impact on HRQoL. Therefore, the impact of comorbid disorders on HRQoL is a point of interest in this study, as the presence of self-perceived high levels of anxiety and depression had the largest impact on patients’ HRQoL scores. First, the study found no association between axis I comorbidities and HRQoL scores except on the physical dimension of the SF-12, although the self-perceived high levels of anxiety and depression on the HADS have been shown to affect HRQoL scores. Only one-third of the sample had an axis I comorbidity diagnosis reported by the physician, while more than half of the sample expressed moderate or severe levels of anxiety and depression on the HADS. As previously reported [28], this discrepancy might reflect an important difference in the methodological approach to measuring comorbidity, in particular psychiatric comorbidity for these patients with substance disorders: clinician-based diagnosis vs. structured interviews*.* Furthermore, it should be noted that the study was conducted in a drug addiction treatment setting, and other prevalence rates could probably be observed in mental health settings. The cooccurrence of SUD and other psychopathologies, especially mental disorders, has been described in previous works [48, 49]. The relationships among these disorders are complex and may take three different forms: SUD may be a primary disorder that determines the development of other disorders; SUD may develop as a consequence of other psychopathological disorders; and SUD and other psychopathological disorders may develop together [50].

The study highlighted that patients with depression according to the HADS had higher impaired HRQoL than those with no depression on the HADS at baseline, but they were also the ones for whom a significantly greater increase in the HRQoL mental domain was observed, particularly during the first 3 months. The beginning of the process of care allowed individuals with a likely dual diagnosis to rapidly reduce accommodating behaviors, thus alleviating the burden of illness. The large impact of self-perceived mental status on HRQoL changes suggests the need not only to perform early systematic psychiatric screening of persons presenting for substance abuse treatment [51] but also to set up more targeted psychological interventions during care to manage emotional problems and reduce anxiety and depression [52].

This study had some limitations that should be considered when interpreting the results. A primary limitation of this study is the small size of the sample. The patients were recruited from French specialty treatment service centers, so these results cannot be generalized to other patients in other countries with different recruitment processes or settings. Moreover, the fact that patients with substance dependence were included prior to publication of the DSM-V and were thus diagnosed in accordance with the DSM-IV criteria limits the applicability of our findings to all patients with SUD, as the DSM-V combines the abuse and dependence criteria into a single SUD [53]. This study is also limited by the fact that we used a generic HRQoL instrument that may not be relevant to patients with SUD due to the specific and different expectations that outpatients may have. However, no specific instrument has yet been developed to assess changes in HRQoL in individuals with SUD [54]. The small R2 of our model suggests that the common HRQoL changes we observed explain only a portion of the variance in change in HRQoL. This finding suggests that other factors not captured in the epidemiologic data, such as coping or other psychosocial adaptations to chronic illness, may be related to changes in HRQoL over time [55]. Future studies should consider this combination, as patients with SUD show a tendency to use dysfunctional strategies when facing problems, and addiction treatment programs should include strategies to reduce such disengagement [56].

However, one of the major strengths of the study is its prospective design. The study provided valuable data on how improvements in the MCS score during the first 3 months of treatment are particularly affected by depressive symptoms at baseline. Such results may require tailored interventions to further screen dual disorders at the beginning of care and to manage depressive symptoms over time. In light of our findings, anxiety and depression screening instruments may be useful in addition to the initial evaluation. Furthermore, as patients with SUD are increasingly treated in ambulatory settings, the factors associated with HRQoL improvements may become more important. Consequently, beyond the health-related aspects of psychological distress and the SUD, it appears necessary to pay attention to issues such as housing, social support, and aspects of life that are meant to enhance individuals’ HRQoL over time. Finally, in this context of outpatient settings, our demonstration of longitudinal associations between health status at baseline and changes in HRQoL indicates that patients are aware of changes in symptoms and daily functioning and can reliably report them on patient reported outcome measure (PROM) instruments. The consideration of HRQoL in clinical practice may prove useful for monitoring the impact of SUD treatment on daily functioning.

## Conclusion

We found substantially lower HRQoL in outpatients with SUD entering care than in the general population, especially in the mental domain. A rapid improvement was seen in HRQoL by month 3, which was then maintained over time. Knowledge of the factors associated with HRQoL changes over time in SUD outpatients is of interest. Consideration of these associated factors, such as occupational status, anxiety and depression, will contribute to the design and planning of comprehensive therapy programs. Treatment approaches considering HRQoL are likely to be more effective in patients with SUD as they track anxiety and depression symptoms. Our findings support the usefulness of both generic HRQoL assessments and mental disorder assessment as complementary objective measurements that increase the clinical understanding of patients receiving SUD treatment.

## Data Availability

Data will not be shared to protect the participants’ anonymity.

## Abbreviations

DSM IV: Diagnostic and Statistical Manual of Mental Disorders, fourth edition
HADS: Hospital Anxiety and Depression Scale
HRQoL: Health-related quality of life
QoL: Quality of life
SF-12: Short-Form 12 questionnaire
SUBUSQOL: SUBstance Users Satisfaction and Quality Of Life
SUD: Substance use disorder
PROM: Patient-reported outcome measure
